# An eHealth ecosystem for stepped and early psychosocial care in advanced lung cancer: Rationale and protocol for a randomized control trial

**DOI:** 10.1016/j.invent.2023.100620

**Published:** 2023-04-03

**Authors:** Cristian Ochoa-Arnedo, Alejandra Arizu-Onassis, Joan C. Medina, Aida Flix-Valle, Laura Ciria-Suarez, Darío Gómez-Fernández, Arnau Souto-Sampera, Isabel Brao, Ramon Palmero, Ernest Nadal, Jesús González-Barboteo, Maria Serra-Blasco

**Affiliations:** a eHealth ICOnnecta't Program and Psycho-Oncology Service, Institut Català d'Oncologia, 08908 L'Hospitalet de Llobregat, Spain; b Psycho-oncology and Digital Health Group, Health Services Research in Cancer, Institut d'Investigació Biomèdica de Bellvitge (IDIBELL), 08908 L'Hospitalet del Llobregat, Spain; c Department of Clinical Psychology and Psychobiology, Universitat de Barcelona, 08035 Barcelona, Spain; d Department of Psychology and Education Sciences, Universitat Oberta de Catalunya, 08018 Barcelona, Spain; e Thoracic Oncology Unit, Department of Medical Oncology, Institut Català d'Oncologia, Hospital Duran i Reynals, Avinguda Gran via 199-203, L'Hospitalet, Barcelona 08908, Spain; f Preclinical and Experimental Research in Thoracic Tumors (PReTT) Group, OncoBell Program, IDIBELL, Avinguda Gran via 199-203, L'Hospitalet, Barcelona 08908, Spain; g Research & Knowledge on Palliative Care Group (GRICOPAL), Bellvitge Institute for Biomedical Research, Barcelona, Spain; h Palliative Care Department, Catalan Institute of Oncology, L'Hospitalet de Llobregat, Spain

**Keywords:** Lung cancer, Psycho-oncology, Psychosocial care, Palliative care, eHealth, Quality of life

## Abstract

**Background:**

Receiving a diagnosis of lung cancer is an emotional event, not least because it is usually diagnosed at advanced stages with limited life expectancy. Although evidence-based educational, emotional, and social interventions exist, they reach few patients and usually when it is too late.

**Objective:**

This project will be carried out in a comprehensive center for cancer care and health research, aiming to study the efficacy, costs, and utility of an eHealth ecosystem to meet the psychosocial needs of patients with advanced lung cancer.

**Method:**

We will enroll 76 patients with advanced lung cancer into an eHealth ecosystem of stepped and personalized psychosocial care for 9 months. These patients will be compared with another 76 receiving usual care in a non-inferiority randomized controlled trial. The following main outcomes will be measured every 3 months: emotional distress, spirituality, demoralization, quality of life, and medication adherence. Secondary outcomes will include symptomatology, health education, cost-utility analyses, usability and satisfaction with the platform, and time to detect emotional needs and provide care. Baseline differences between groups will be measured with the Student *t-*test or chi-square test, as appropriate. We will then compare the main outcomes between groups over time using multilevel linear models, report effect sizes (Hedges' *g*), and assess non-inferiority. The cost-utility of both interventions will be considered in terms of quality adjusted life years and quality of life given the costs of providing each treatment.

**Discussion:**

This randomized controlled trial should provide new evidence on the efficacy and cost-utility of an eHealth ecosystem to deliver personalized and timely psychosocial care to patients with advanced lung cancer.

**Trial registration:**

ClinicalTrials.gov ID “NCT05497973”.

## Highlights

• Around 60% of patients with advanced lung cancer suffer clinically significant psychological distress
• First digital early stepped psychosocial care tailored to cover lung cancer patients’ necessities
• The ecosystem is based on four levels: distress monitoring, health literacy peer support and online psychotherapy
• This eHealth psychosocial program may offer a compliant and cost-effective person-centred care

## 1 Introduction

Lung cancer affects >2 million people worldwide each year and has a 5-year patient survival of only 13 % (Sung et al., 2021). Estimates in the European Union indicated that there had been 318,327 new cases in 2020, marking the lung as having the highest mortality of 20.4 % of all cancer-related deaths (European Union, 2021). Affected patients also frequently encounter severe symptoms that show a variable and erratic course over time and make the illness course highly unpredictable. Consequently, these patients tend to have a very poor quality of life (QoL), especially in advanced stages (Presley et al., 2020), and up to 63 % experience clinically significant psychological distress (Graves et al., 2007; Lv et al., 2021). Indeed, a recent survey of over 9000 patients with lung cancer indicated that most functional limitations were due to pain (68.6 %) or emotional distress (48.3 %) that remained untreated in 32.5 % (Presley et al., 2020). Psychosocial care is generally inadequate, with many patients having no possibility of receiving appropriate care. Given that this distress does not remit over time (Aubin et al., 2022), it is urgent that we improve screening, offer close and intensive follow-up, and refer to adequate and accessible services early.

Palliative care refers to the medical relief typically offered to people living with incurable illnesses, and for those with advanced lung cancer, it typically focuses on common physical symptoms like fatigue, dyspnea, and chronic pain (Rajapakse, 2021). Over recent decades, however, the value of addressing a person's psychosocial and spiritual needs has also been recognized. American and European societies for medical oncology now advocate the integration of early psychosocial palliative care in standard oncology practice for patients with metastatic or advanced disease. This decision has been supported by the results of a recent meta-analysis (Fulton et al., 2019), which demonstrated that palliative interventions with physical and psychological aspects can benefit a patient's short-term QoL and overall symptom burden. Evidence suggests that a patient's emotional distress is associated with not only poorer QoL but also lower adherence to treatment and the adoption of unhealthy lifestyles (Jansen et al., 2017). Despite this growing evidence, emotional distress is not always considered in palliative care, leaving many patients with undetected and unresolved psychosocial needs.

Distress is a common and frequently ignored psychosocial dimension in patients with advanced cancer, but evidence suggests it can be improved by including a consideration of QoL rather than survival in isolation (Reale et al., 2020). QoL is a broad concept that includes emotional well-being, self-care, and performing usual daily activities. It has been evidenced that patients with an enhanced sense of psycho-spiritual well-being can cope more effectively with a terminal illness and find meaning in the experience (Lin and Bauer-Wu, 2003). Indeed, several studies have found a relationship between greater spirituality and both QoL (Steinhauser et al., 2017) and emotional well-being (Moyano et al., 2019) during palliative care. By contrast, demoralization appears to be an important risk factor for patients' distress that negatively affects spiritual well-being (Liu and Hsiao, 2019). This is important because it reaches clinically significant levels in 13 %–18 % of patients with advanced cancer (Robinson et al., 2015). Other sources of distress include disinformation, poor health literacy, and issues with family communication. Nevertheless, each of these has shown improvement when addressed with psychosocial interventions (Graves et al., 2007).

Cancer-related distress affects both the emotional and physical statuses of patients, and together with inadequate social support, represent a major contributor to treatment adherence in patients with lung cancer (Liu et al., 2022). Similarly, depression is independently associated with not only treatment adherence but also with a poorer prognosis (Arrieta et al., 2013). Any holistic psychosocial intervention for patients with lung cancer should also consider health literacy, which itself is associated with medication adherence (Gönderen Çakmak and Uncu, 2020).

Despite the growing and evident health benefits of comprehensive palliative care interventions, various barriers exist to the implementation of psychosocial care in hospital settings. The most frequent of these are the failure to detect needs early, the long waiting lists, and the restricted mobility of many patients (Travado et al., 2017). To improve the implementation of psychosocial care, efforts have focused on improving its accessibility and efficiency by introducing early and tailored psychosocial interventions that are both customized to the patient and cost-efficient for service providers (Bekelman et al., 2016). It is here that information and communication technologies (ICTs) are now playing a pivotal role in health care (eHealth), offering a feasible solution that can ensure continuity of their care in patients with advanced cancer (Kroll et al., 2021). The use of eHealth can improve the monitoring of warning signs; facilitate better communication with professionals; and leverage clinical treatments that are cheaper and more accessible than traditional modalities (Penedo et al., 2020). Although this appears to apply to palliative care (Widberg et al., 2020), the few studies comparing eHealth with usual psychosocial interventions have found mixed efficacy in palliative settings (Escriva Boulley et al., 2018).

This clinical trial is preceded by a European-funded project (ONCOMMUN; https://oncommun.eu/) that developed an eHealth ecosystem for patients with breast cancer. This project aimed to facilitate early psychosocial care for patients with breast cancer via an online, stepped program implemented at the Institut Català d'Oncologia (ICO) through the ICOnnecta't program (Ochoa-Arnedo et al., 2020). The tiered intervention has four levels, starting with screening and monitoring of psychosocial needs and offering more intensive interventions as patients need them, and the last level is group psychotherapy. This has shown promising early results in breast cancer (Ciria-Suarez et al., 2022; Medina et al., 2022) and the potential to address the psychosocial needs in lung cancer (Graves et al., 2007). It is anticipated that a stepped, tailored, psychosocial eHealth intervention based on this model will detect distress in patients with lung cancer quickly and facilitate the provision of personalized solutions through psychological counseling, health literacy, and social support in the community. In turn, these can impact variables like demoralization, spirituality, and medication adherence.

### 1.1 Aims and hypotheses

The main objective of the project is to implement and evaluate the efficacy of a stepped online ecosystem for detecting, following, and addressing the psychosocial needs of patients with lung cancer and to compare those with usual face-to-face treatment. We aim to detect cases at risk of significant emotional distress and decrease the time to access appropriate psychosocial care when using eHealth and usual face-to-face care.

Comparisons between the two interventions will consider the effects on ameliorating the psychosocial consequences of lung cancer and its treatment on emotional distress, demoralization, QoL, and medication adherence, including consideration of the role of spirituality and patient perception of their health education. The cost-utility of each intervention will then be analyzed in terms of quality adjusted life years (QALYs), considering use of additional health services, consumption of psychotropic drugs, sick leave durations, professional salaries, and infrastructure and transportation costs. We will then measure perceived satisfaction and usability of the eHealth ecosystem. Finally, the cases at risk of emotional distress will be detected earlier than treatment as usual. Consequently, using new technologies, we will decrease the time to access appropriate psychosocial care.

Considering previous results, the literature reviewed, and the objectives set forth, we hypothesized that two psychosocial treatments will improve emotional distress, demoralization, QoL, and medication adherence. Moreover, these improvements will be greater in patients with worse psychosocial results at baseline. For patients with a better baseline status, we expect the psychosocial interventions to exert a buffering effect despite disease progression. The eHealth ecosystem will obtain results equivalent to usual care for emotional distress, demoralization, QoL, and medication adherence. Spirituality at baseline, or its augmentation by psychosocial interventions, will predict better outcomes for emotional distress, demoralization, QoL, and medication adherence. Health education will improve markedly in the group allocated to the eHealth intervention. Compared with usual care, eHealth will have a superior cost-utility in terms of QALYs. Finally, users will be satisfied with the eHealth ecosystem and will perceive it as easy to use. The eHealth ecosystem will significantly improve the time to detection, intervention, and resolution of clinically significant psychosocial need compared to usual care.

## 2 Material and methods

### 2.1 Study design

This study proposes a randomized non-inferiority trial of an eHealth ecosystem compared to usual psychosocial care. The main outcomes will be assessed at baseline (T0), 3 months from T0 (T1), 6 months from T0 (T2), and 9 months from T0 (T3). Therefore, the design comprises two treatment conditions and four assessments. We chose the non-inferiority based on a literature review that indicated mixed and inconsistent results when comparing eHealth and face-to-face psychosocial interventions in cancer (Merluzzi et al., 2019; Singleton et al., 2022; Wang et al., 2020; Xu et al., 2019). Therefore, we do not hypothesize that our eHealth ecosystem will be more efficacious than usual care, but instead, aim to show that it is at least not inferior in terms of efficacy and efficiency. The methodology will adhere to the principles of Responsible Research and Innovation (RRI, European Commission, Directorate-General for Communication, 2013).

### 2.2 Participant recruitment and procedure

**Fig. 1:**
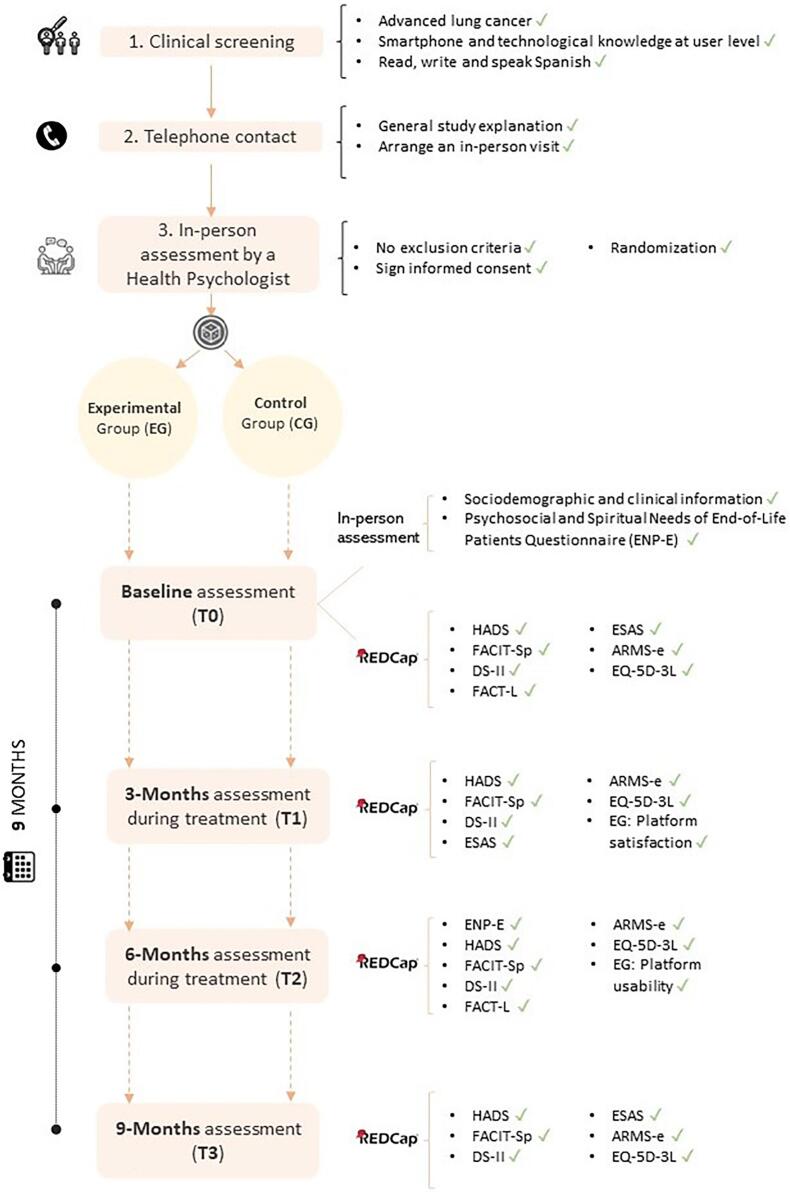
Flowchart of the recruitment process and study follow-up assessments. See questionnaires details below (2.5 Measures).

Participants will be recruited through different healthcare units (e.g., the Functional Lung Unit and the General Palliative Care Outpatient Service at ICO). When clinicians from these services identify a patient who meets the inclusion criteria, they will contact researchers from ICO's eHealth program, who in turn, will contact the patient (See Fig. 1 for details of recruitment).

### 2.3 Inclusion and exclusion criteria

Individuals agreeing to participate will be screened to verify whether they meet all the following inclusion criteria: age ≥ 18 years; recent diagnosis (<3 months) of advanced lung cancer (stage III–IV); internet access and digital experience; and ability to read, write, and speak Spanish. The exclusion criteria will include current major depressive episodes, risk of self-harm, active psychotic symptoms, and substance abuse.

### 2.4 Timeline

At the baseline visit, researchers will explain the study and obtain informed signed consent. They will then introduce the patient to REDCap, a secure web platform for building and managing online databases and surveys (https://www.project-redcap.org/). After checking eligibility at baseline, patients will be randomly allocated to one of the two treatment arms by REDCap, using simple randomization. The clinician-administered face-to-face questionnaires will be administered to all patients at this baseline evaluation (T0), while REDCap will send self-administered measures by e-mail at baseline (T0), 3 months (T1), 6 months (T2), and 9 months (T3) (Fig. 1).

### 2.5 Measures

Sociodemographic and clinical data regarding cancer characterization will be obtained from two sources: ICO's computerized clinical history system (Patient Care Service, SAP https://www.sap.com/index.html) and semi-structured interviews using the Psychosocial and Spiritual Needs of End-of-Life Patients Questionnaire (ENP-E) (Mateo-Ortega et al., 2019). The ENP-E is a hetero-administered clinical instrument with three subscales: evaluation of psychosocial needs (12 items), exploration of worries (1 item), and external signs of emotional distress (1 item). A score ≥ 28 indicates moderate to severe need and recommends assessment for specialized intervention.

#### 2.5.1 Primary outcomes

Hospital Anxiety and Depression Scale (HADS): Assessed emotional distress through 7 anxiety items (HADS-Anxiety) and 7 depression items (HADS-Depression). The range of scores is 0–21 for each subscale, and 0–42 for the overall questionnaire. An overall score ≥ 10 is indicative of moderate emotional distress and ≥ 16 of high emotional distress (Carmen Terol-Cantero et al., 2015).

Functional Assessment of Chronic Illness Therapy-Spiritual Well-Being (FACIT-Sp): Spirituality will be assessed through the Spanish version of the FACIT-Sp (Peterman et al., 2002). This instrument assesses three dimensions of spirituality through two subscales: 1) meaning and peace (8 items), and 2) faith (4 items). The overall score ranges between 0 and 48, between 0 and 32 for the first subscale, and 16 for the second. Higher scores are indicative of greater spiritual well-being.

Demoralization Scale – abbreviated version (DS-II): Patient's demoralization will be assessed through the Spanish version of DS-II (Belar et al., 2019). The DS-II is composed of 16 items, 8 for each of its two subscales: 1) meaning and purpose, and 2) coping. Scores <10 indicate no demoralization, between 10 and 19 moderate demoralization, and > 20 severe demoralization.

Functional Assessment of Cancer Therapy – Lung Cancer (FACT-L): to measure the QoL of LC patients, the Spanish adaptation of the FACT-L will be used (Cella et al., 1998). The FACT-L is an instrument derived from the general cancer QoL questionnaire, FACT-G, with an added subscale specific to LC and its symptoms. It has 36 items organized into 5 subscales: 1) physical well-being, 2) functional well-being, 3) social/family well-being, 4) emotional well-being and 5) LC. Higher scores correspond to a better QoL.

Adherence to Refill and Medication Scale (ARMS-e): Medication adherence will be assessed trough the Spanish version (González-Bueno et al., 2017) of ARMS-e, which is a valid and reliable scale for patients with a chronic disease. This instrument consists of 12 questions: 8 are focused on the patient's consistency in taking medication appropriately and 4 on their proper collection. Lower overall scores correspond to better adherence.

#### 2.5.2 Secondary outcomes

Edmonton Symptom Assessment System (ESAS-r): Patients' symptomatology will be controlled through ESAS in its revised version, adapted and validated in Spanish (ESAS-r) (Carvajal et al., 2013). ESAS-r is an instrument that is commonly used both in palliative care and in advanced cancer situations. It has 10 visual numerical scales that assess physical and psychological symptoms. Higher scores are indicative of higher pain/distress. The burden and severity of symptoms may affect other variables analyzed in the study and.

Health education: will be assessed using a visual analogue scale (VAS, 0–10) scoring patients' satisfaction with the health information received during their cancer journey, both physically and emotionally.

##### 2.5.2.1 Cost-utility analyses

European Quality of Life Scale (EQ-5D-3L): The EQ-5D, which provides a measure of Health-Related QoL (HRQoL), is helpful for the evaluation of the cost-utility of health interventions (Kennedy-Martin et al., 2020). It will be administered in its Spanish adapted and validated version (Badia et al., 2001). This instrument is composed of three sections: 1) descriptive system, 2) visual analogue scale (VAS), and 3) social value index. The value index ranges from 1 (best health) to 0 (death) and is used alone or in combination with life years for the calculation of Quality Adjusted Life Years (QALYs).

Use of medical care and psychotropic medication: These variables will be assessed through SAP by collecting the number of patients' visits with each health professional (e.g., oncologist, emergency department, social worker, psychiatrist, psychologist, nurse), as well as their prescribed psychotropic medication (i.e., drug, dose, and schedule). Costs will be calculated according to national or institutional rates.

Time off work: the number of sick leave days for each patient will be evaluated through SAP. Costs will be estimated according to national rates.

Professional salaries: The costs of hours that professionals dedicate per week to each treatment arm will be assessed through SAP and institutional rates.

Infrastructure costs: the costs per patient per month required for the development and maintenance of the online platform in the case of the EG, and these costs applied to the hospital infrastructure to ensure face-to-face visits in the case of the CG, will be assessed through each provider (EG: technology provider; CG: hospital management).

Transport costs: an estimate of the cost per patient in using a given type of transport will be obtained based on official mobility data provided at the government level for the metropolitan area of Barcelona.

##### 2.5.2.2 Satisfaction with and usability of the digital platform (only for patients included in the experimental group -EG- ICOnnecta't)

Satisfaction: will be assessed using a visual analogue scale (VAS, 0–10) in the EG patients scoring their satisfaction with the online platform used during treatment.

Usability: will be assessed using a VAS (0−10) in the EG patients scoring their perception of the usability of the online platform used during treatment.

##### 2.5.2.3 Early detection of patients at risk of emotional distress and time to receive appropriate psychosocial care

**Fig. 2:**
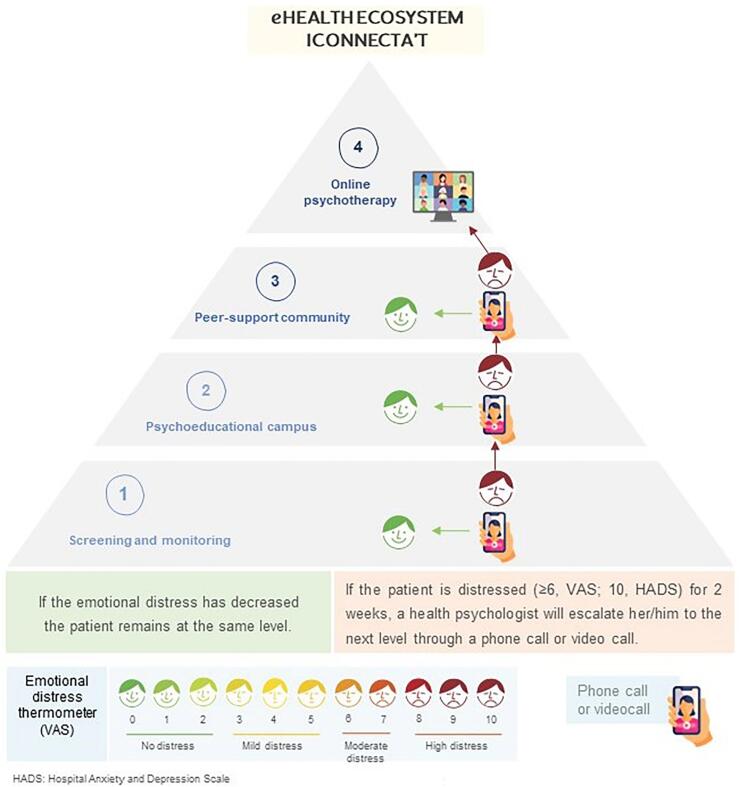
Stepped digital psychosocial intervention ICOnnecta’t.

Well-being - emotional discomfort screening: it will be evaluated through an emotional state thermometer with a response system in VAS format, which will be scheduled weekly on the ICOnnecta't platform (see Fig. 2).

The number of detected clinical cases: in the EG this will be obtained through the number of potential clinical cases recorded throughout treatment (VAS ≥6). In CG it will be obtained through the number of clinical cases recorded in the follow-up of face-to-face visits or if the patient reports a worsening by telephone and the visit is brought forward to confirm if it is a clinical case (VAS ≥6).

Waiting time to provide psychosocial intervention: In the EG, the time between the occurrence of a potential clinical case (VAS ≥ 6) and the start of psychosocial intervention will be assessed. The CG will assess the time between the face-to-face screening visit that confirms the clinical case and the next face-to-face visit.

Percentage of resolved psychosocial cases: in both groups it will be obtained through the number of clinical cases that are resolved.

### 2.6 Interventions

#### 2.6.1 The eHealth ecosystem, ICOnnecta't

ICOnnecta't is a stepped-care psychosocial intervention that comprises four levels staggered by psychosocial complexity (see Fig. 2). In this way, it aims to provide progressively adjusted psychosocial care starting with the least intensive and complex intervention first (Hutchison et al., 2006). Through the systematic monitoring of specific clinical variables (Level 1, i.e., emotional distress), the patient's psychosocial status can be established to decide whether they should move to a more intensive and complex step (Lutz, 2003). The intervention levels of ICOnnecta't are based on those of other published interventions that use a stepped approach, and they aim to manage emotional distress in the cancer population. Four levels of care are usual: that range from basic psychoeducation (ICOnnecta't level 2) to intensive psychotherapy (ICOnnecta't level 4) (Braamse et al., 2016; Butow et al., 2015; Hauffman et al., 2020; Hutchison et al., 2006; Krebber et al., 2016; Kusch et al., 2022; Rissanen et al., 2015). Some authors recommend incorporating peer support communities into stepped interventions to promote wellness, self-care habits, and connectedness (Butow et al., 2015; Hauffman et al., 2020; Hutchison et al., 2006; Ramchand et al., 2017).

All patients will join the program at level 1 and, each time they step up, will retain access to the previous step (Chan et al., 2013). All patient journeys in the stepped intervention will be guided by a lead health psychologist with expertise in psycho-oncology. Patients can be referred to psycho-oncology, psychiatry, or social work departments, as necessary. The detailed interventions at each level, together with the escalation protocol, are based and adapted on the protocol used for breast cancer (Ochoa-Arnedo et al., 2020), and these are detailed below.

##### 2.6.1.1 Level 1: screening and monitoring

Screening of potential emotional distress and monitoring of physical symptoms through a mobile application (ICOnnecta't App). Patients can spontaneously communicate their mood through a VAS emotional thermometer. In addition, a health psychologist will program a weekly assessment with the same thermometer, with a score ≥ 6 (10 indicating maximum distress) triggering a video consultation or phone call to assess the distress and explore any specific psychosocial needs. These sessions are also intended to accompany the patient throughout their cancer journey. Before a session, patients complete the HADS, and if both the HADS (≥ 10) and the psychologist's impression confirm clinically significant distress, the patient will be escalated to the next level. This adaptation of a stepped-care program used in oncology will be followed for escalation to the subsequent levels (Jansen et al., 2017).

##### 2.6.1.2 Level 2: campus: psychoeducation and health education

The ICOnnecta't Campus is an educational platform that can be accessed via Moodle. It allows patients to consult videos and online resources developed by health professionals and based on patient feedback. The campus therefore contains scientifically validated information related to the diagnosis and treatment of advanced oncological disease, organized into different thematic blocks that represent the usual challenges involved with an advanced oncological diagnosis (Graves et al., 2007). The key areas are as follows: (a) The medical aspects of lung cancer in 14 resources (i.e., common symptoms, medical treatments, and side effects; (b) Emotional aspects, with eight resources about emotional responses to diagnoses and treatment; (c) Personal relationships, with four resources addressed to family members and caregivers about the role of the family in treatment and how they can aid the patient during the process; d) Healthy habits, comprising 28 resources about exercise, healthy eating models, rest and self-care; and e) Spiritual, comprising three resources about spiritual worries, shared decision-making, and advance directives. The patient will remain at this level for two weeks. If distress does not decrease on the thermometer (score ≥ 6), a HADS and video consultation or phone call will be scheduled to assess the appropriateness (HADS score ≥ 10) of moving to the next level of care.

##### 2.6.1.3 Level 3: community psychosocial support

The service provides a safe online community where patients can anonymously share potential concerns related to the oncological process, resolve doubts and health issues, share experiences, or discuss issues related to the disease. This private social network contains several discussion forums related to advanced lung cancer and its possible implications, and they parallel the thematic blocks of ICOnnecta't Campus (level 2). It is supervised by a health psychologist who will provide a professional response with the support of other specialist members of the oncology team (e.g., psycho-oncologist, nurse, psychiatrist, and social worker). As in the previous level, the patient remains in this level for two weeks and is only advanced to level 4 if distress remains high (thermometer score ≥ 6), and a subsequent HADS score, and video or phone consultation indicate this is appropriate.

##### 2.6.1.4 Level 4: online group psychotherapy

In the final level, patients are offered online group psychotherapy led by a clinical psychologist with expertise in psycho-oncology. This comprises eight weekly 90-min sessions that use the meaning-centered group psychotherapy approach developed by Breitbart et al. (2015). Patients at this level remain on a waiting list until four to eight users become available for the group sessions.

#### 2.6.2 Treatment as usual - psychosocial face-to-face treatment

Patients in the control group will receive standard in-person psychosocial care delivered by a clinical psychologist. This will consist of an average of seven individual sessions, lasting 45–60 min each, with an inter-session spacing of 2–3 weeks. The clinical psychologist will provide the intervention based on individual meaning-centered psychotherapy for advanced cancer patients (Breitbart et al., 2012), aiming to help maintain or enhance the sense of meaning, peace, and purpose in the lives of patients with advanced cancer. Patients in this treatment arm will also have access to the educational materials of level 2 of the eHealth intervention, which will be provided in a freely accessible web portal for all patients and their families.

### 2.7 Power analysis

The sample size has been estimated using R software, setting a non-inferiority margin of 5 points on the HADS (Vaganian et al., 2020) with a power of 80 % and a one-tailed α of 2.5 %. Assuming a dropout rate of 25 %, we require 152 participants (76 per branch) to ensure a 95 % bilateral confidence interval that excludes the non-inferiority threshold.

### 2.8 Data protection and ethics compliance

Two databases will be created. The first will turn participants' personal identification data (e.g., names, patient ID) into an alphanumeric code that will be saved on an external hard drive and stored in a locked cabinet within the principal investigator's office. The second will use these alphanumeric codes to record all data collected for analysis, and it will be stored in a REDCap repository compliant with the General Data Protection Regulation. This procedure allows us to perform the anonymous analysis. The data will be downloaded monthly from the online questionnaire system used for the evaluations, coded in both databases, and backed up on a second encrypted external hard drive.

### 2.9 Planned statistical analysis

Descriptive analyses will be performed using Student t or chi-squared tests, as appropriate. This will include assessment of sociodemographic and clinical variables at baseline, together with any pre-treatment differences between groups. Intention-to-treat analyses will then be performed through multilevel linear models to compare both groups on each primary outcome: emotional distress (HADS), demoralization (DS-II), QoL (FACT-L), and medication adherence (ARMS-e).

The modeling process will be parsimonious and guided by fit indexes, starting with a model using the time and type of therapy as fixed predictors. Then, random effects will be tested, and the interaction between time and type of therapy, the eHealth step reached, symptomatology (ESAS-r), and spirituality (FACIT-Sp) will be progressively entered as predictors. The estimation method will be maximum likelihood. Effect sizes (Hedges' g) will be reported for the primary outcomes and non-inferiority will be assessed for the HADS by comparing the means of both groups at T2 and T3. Additional multilevel linear models will be run to assess changes in health education (VAS) between groups, according to the same procedures. As for the cost-utility analyses, the EQ-5D will be used to compute the QALYs together with associated costs for medical care, psychotropics, sick leave, professional salaries, infrastructure, and transportation.

Descriptive analyses will also be conducted for platform's satisfaction and usability (VAS), as well as for participants reaching each step in the eHealth group. Mean differences in the number of clinical cases detected (according to the emotional thermometer), waiting time to provide care, and number of clinical cases solved will then be compared between groups by Student *t*-tests. For the reporting of results, 95 % confidence intervals will be provided. Analyses will be performed using IBM SPSS version 24 (IBM Corp., Armonk, NY, USA) and R software, with support provided by the biostatistics department of IDIBELL.

## 3 Discussion

The current protocol describes a randomized controlled trial intended to examine whether a stepped-care, eHealth-based psychosocial intervention can effectively detect and address the psychosocial needs of patients with advanced lung cancer. We plan to conduct an open-label randomized controlled trial in two groups: one receiving an individualized online stepped-care psychosocial intervention, and the other group receiving an average of seven face-to-face visits with a clinical psychologist.

Patients with lung cancer are mostly diagnosed at advanced disease stages and have minimal prospect of cure. Such a scenario produces stress and psychological trauma for many patients, which in turn, affects their QoL (Liegey Dougall et al., 2017). Given that effective palliative care should improve the QoL of patients and their families, tackling their psychosocial needs in an efficient manner should be among the top priorities of comprehensive palliative care. Health education (Yu et al., 2022) and psychosocial care (Jansen et al., 2019) can both mitigate the emotional distress of a diagnosis and improve QoL among patients with lung cancer. However, the limited access to such care presents an important barrier (Travado et al., 2017).

The use of eHealth can overcome barriers requiring physical attendance at health centers with symptoms of fatigue, dyspnea, or pain (Rajapakse, 2021). As ICTs have emerged to deliver psychosocial interventions reliably to vulnerable patients with advanced cancer (Penedo et al., 2020), clinicians and researchers have begun to study techniques to deliver palliative interventions in an eHealth format (Chua et al., 2019; Gómez-Batiste et al., 2014). However, existing research has usually centered on physical symptoms, not the emotional and spiritual concerns of patients. Furthermore, patients benefit most from psychosocial interventions when they have elevated baseline emotional distress (Jansen et al., 2019; Medina et al., 2022; Nissen et al., 2021), stressing the importance of individualized strategies that allocate resources to patients with at-risk profiles.

The online, stepped-care ecosystem we propose has significant potential for therapeutic application in palliative care settings, with this being the first study in advanced lung cancer to consider a predominant psychosocial approach. In the proposed intervention, levels of care are scaled by the complexity and technological integration of screening and follow-up, based on evidence that psychosocial interventions are emerging as a solution to increase service coverage and improve efficiency.

Implementing an online care system that monitors and adapts to the changing needs of the patients at the end-of-life will promote personalized and responsive attention. If cost-utility is proven, health providers may cover more psychosocial needs. In addition to reaching more patients, the proposed eHealth ecosystem should contribute to improved patient empowerment, health literacy, and involvement in actions designed to improve health. By promoting greater access, this program will also promote equity (Ciria-Suarez et al., 2022) during the process of improving QoL.

The first limitation of this study is its focus on patients with advanced lung cancer. This may make it difficult to generalize the current findings to other cancer populations. However, given that it will cover a broad range of psychosocial needs that are shared by all cancer patients, it should be able to serve as a starting point for developing other end-of-life care programs, regardless of the underlying pathology. Other methodological weaknesses include the high risk of dropout and missing data with online questionnaires, especially in this delicate cancer population. To diminish this risk, we have shortened the 1-year follow-up used in similar programs to just 9 months. Also, a health psychologist will closely follow all included patients and help them to complete the online questionnaires by phone when needed. The online questionnaires are also structured so that they begin with the primary outcomes in anticipation that this might facilitate their completion, and in any case, they have been designed to last a maximum of 20 min. Finally, although online treatments are increasingly considered acceptable, age and digital literacy still dictate access to ICT, and by association, the study. To improve digital literacy, the first face-to-face visit will include a digital welcoming session and we will provide a technical support service to all patients, including a video tutorial about how to use the ICOnnecta't application.

### 3.1 Conclusions

This proposed work aims to evaluate the non-inferiority of an eHealth ecosystem that provides psychosocial health care to patients with advanced lung cancer. The ecosystem will be divided into four steps of increasing complexity, ranging from prevention and health promotion to community interventions and psychotherapy. If our objectives are achieved, this research could generate major advances toward a truly integrated model of eHealth psychosocial care in oncology and other end-of-life settings. We believe our findings could serve as a turning point in providing sustainable person-centered care using internet devices.

## CRediT authorship contribution statement

COA designed the study. MSB wrote the first draft of the manuscript. COA, AA, JCM, AFV, LCS, and JGB critically revised the manuscript. All authors have consistently contributed with feedback on the study design, read and revised the manuscript, and approved the submitted version.

## Declaration of competing interest

The authors declare that they have no known competing financial interests or personal relationships that could have appeared to influence the work reported in this paper.
